# Supplementary material for: Perinatal High-Salt Diet Induces Gut Microbiota Dysbiosis, Bile Acid Homeostasis Disbalance, and NAFLD in Weanling Mice Offspring
Source: Nutrients. 2021 Jun 22;13(7):2135. doi: 10.3390/nu13072135 (PMC8308454; doi:10.3390/nu13072135)
Supplement: Supplementary file 1 [file nutrients-13-02135-s001.zip › nutrients-1153542-supplementary.pdf]

Table S1. The compositions and formulas of normal diet and high salt diet

**Class description**	**Ingredient (g/kg Diet)**	**Normal Diet**		**High Salt Diet**	
		**g**	**Kcal**	**g**	**Kcal**
Protein	Casein,30 mesh	200	800	200	800
Protein	L-cystine	3	12	3	12
Carbohydrate	Corn Starch	397	1589	397	1589
Carbohydrate	Maltodextrin 10	132	528	132	528
Carbohydrate	Sucrose	100	400	100	400
Fiber	Cellulose	50	0	50	0
Fat	Soybean Oil	70	630	70	630
Anti-oxidant	t-butylhyroquinone	0.014	0	0.014	0
Mineral	Mineral Mix S10022G	35	0	35	0
Vitamin	Vitamin Mix V10037	10	40	10	40
Vitamin	Choline Bitartrate	2.5	0	2.5	0
	Sodium Chloride	0	0	39.98	0
Total		999.514	3999	1039.49	3999
Component		**g (%)**	**Calorie (%)**	**g (%)**	**Calorie (%)**
Protein		20	20		20
Carbohydrate		63	63		63
Fat		7	16		16

Table S2. Primer sequences.

Gene	Accession Number	Sequences
Cyp7a1	NM_007824.3	F-5‘-tgatcctctgggcatctcaagcaa-3'<br>R-5‘-agctcttggccagcactctgtaat-3'
Gapdh	NM_008084.3	F-5‘-acccagaagactgtggatgg-3'<br>R-5‘-cagtgagcttcccgttcag-3'
Srebp1c	NM_001358314.1	F-5‘-ggagccatggattgcacatt-3'<br>R-5‘-gcttccagagaggaggccag-3'
Fas	NM_007988.3	F-5‘-aagttgcccgagtcagagaacc-3'<br>R-5‘-atccatagagcccagccttccatc-3'
ACC	NM_133360.2	F-5‘-aacatccccacgctaaacag-3'<br>R-5‘-ctgacaaggtggcgtgaag-3'
Il6	NM_001314054.1	F-5‘-tgatggatgctaccaaactgga-3'<br>R-5‘-tgtgactccagcttatctcttgg-3'
Tnf	NM_013693.3	F-5‘-tagcccacgtcgtagcaaac-3'<br>R-5‘- acaaggtacaacccatcggc-3'
Ocln	NM_008756.2	F-5‘-ttgaactgtggattggcagc-3'<br>R-5‘- caagataagcgaaccttggcg-3'
Tjp1	NM_009386.2	F-5‘-gatgtttatgcggacggtgg-3'<br>R-5‘-cattgctgtgctcttagcgg-3'
Nr0b2	NM_011850.3	F-5‘- tcctcttcaacccagatgtgc-3'<br>R-5‘-agacttcacacagtgcccag-3'
Nr1h4	NM_001163700.1	F-5‘- tggctgaatgtatgtatacaggttt-3'<br>R-5‘-cagcgtgctgcttcacattt-3'
